# Abnormal Expression of c-Myc Oncogene in NK Cells in Patients with Cancer

**DOI:** 10.3390/ijms20030756

**Published:** 2019-02-11

**Authors:** Gulnur K. Zakiryanova, Elena Kustova, Nataliya T. Urazalieva, Emile T. Baimuchametov, Narymzhan N. Nakisbekov, Michael R. Shurin

**Affiliations:** 1 Al-Farabi Kazakh National University, Almaty 050040, Kazakhstan; 2 Laboratory of Immunology, Scientific Center of Pediatric and Children Surgery, Almaty 050060, Kazakhstan; lenbush@list.ru (E.K.); docnat69@mail.ru (N.T.U.); 3 Kazakh Research Institute of Oncology & Radiology, Almaty 480072, Kazakhstan; emil.onco@mail.ru; 4 Joint Use Center, Atchabarov Scientific Research Institute of Fundamental and Applied Medicine, Asfendiyarov Kazakh National Medical University, Almaty 050000, Kazakhstan; nno.niifpm@kaznmu.kz; 5 Departments of Pathology and Immunology, University of Pittsburgh Medical Center, Pittsburgh, PA 15213, USA

**Keywords:** NK cells, c-myc, MAPK, lung cancer, gastric cancer

## Abstract

Natural killer (NK) cells have received a lot of attention in recent years for the roles they play in immunity and particularly in antitumor immune responses. Although defects in NK cell functions are recognized as important mechanisms for immune evasion of malignant cells, molecular pathways regulating NK cell dysfunction and exhaustion in cancer are largely unknown. Here we tested whether the c-myc proto-oncogene, known to promote cell proliferation, growth, differentiation, and apoptosis by regulating the expression of numerous target genes, may be involved in the mechanism of NK cell abnormalities in patients with lung and gastric cancer. Analysis of c-myc mRNA and protein expression in peripheral blood NK cells, mitogen-activated protein kinase (MAPK) activity, cell cycle, and cell longevity revealed a significantly decreased expression of c-myc mRNA and protein and mitotic arrest of NK cells in different phases of cell cycle. In addition, a significant decrease of NK cell death was also detected. These data allow the suggestion that defects of NK cell-mediated tumor surveillance may be associated with disturbed c-myc expression in NK cells in cancer patients. A better understanding of the mechanisms of NK cell dysfunction in cancer will help in the NK cell-mediated therapeutic eradication of primary and metastatic cancer cells and prolong patient survival.

## 1. Introduction

The immune system has a tremendous capacity for defense mechanisms surveilling the appearance, development, and progression of malignant tumors. The activity of such innate mechanisms for battling tumors in their earliest stages has been recently demonstrated using intravital imaging to track the fate of mouse skin epithelium burdened with varying numbers of activated Wnt/beta-catenin stem cells [1]. The results revealed unanticipated plasticity of the adult skin epithelium when faced with mutational and non-mutational insult: oncogene-induced tissue aberrancies were all corrected with the active elimination of mutant cells [1]. However, clinical and experimental data demonstrate that cancers can escape immunosurveillance and immune eradication by evolving a variety of pathophysiological mechanisms, which block, disable, or polarized antitumor immunity. The development of the tumor immunoenvironment that supports tumor expansion, growth and progression (formation of metastasis) is a key mechanism of carcinogenesis; thus, its blocking or disruption is a key goal of modern biological therapeutics. 

Interestingly, simultaneous single-cell analysis of the lung cancer material, noninvolved lung tissue and blood cells has shown that the ‘initial’ and ‘terminal’ clinical stages of the disease do not really differ in their immunological characteristics [2]. Multiple immune defects detected in the late-stage tumor lesions have also been revealed in stage I tumors. It is important to note that the observed pathoimmunological changes have been seen both in the tumor tissue and peripheral blood [2]. Thus, it is conceivable to speculate that at least some of immune defects in cancer patients may develop independently of the stage of the tumor and that these defects are not likely the result of tumor growth. Analysis of quantitative differences in immune cell types between breast tissues from normal donors and those from women with benign breast disease who either subsequently developed cancer or remained cancer-free, revealed similar alterations of the immune system in the earliest stages of breast carcinogenesis [3]. Similarly, investigation of spontaneous tumor in a genetically defined mouse model of pancreatic ductal adenocarcinoma demonstrated a prominent leukocytic infiltration even around the lowest grade preinvasive lesions with immunosuppressive cells dominated the early response and persisted through the invasive cancer [4]. Moreover, effector T cells were scarce in preinvasive lesions, seen in only a subset of advanced cancers and displayed no evidence of activation. Thus, in contrast to the hypothesis that an early "elimination phase" of cancer immune surveillance is finally overwhelmed by a growing tumor, these and other data may suggest that the effective tumor immunity may be undermined from the very beginning. This unusual plasticity of the immune system may provide new tools and new avenue for characterization and understanding the role of immune cells and immune factors in formation of premalignant niches and successful survival of newly appeared neoplastic cells at specific locations. The incompletely understood dynamics of cancer immunosurveillance hampers efforts to develop highly efficient immunotherapy of cancer. 

Assuming that the defects in the immune system may, at least in certain cases, precede the appearance of cancer, we hypothesized that proto-oncogenes in immune cells may also be associated with immune defects in cancer [5]. For example, the c-Myc proto-oncogene is frequently activated in a diversity of malignant cells and plays an important role in cellular differentiation, proliferation, apoptosis and cell cycle progression [6]. Recently, for the first time, we estimated the c-Myc expression in peripheral blood NK cells from patients with cancer: c-Myc mRNA expression, as well as expression of another proto-oncogene c-kit, was significantly decreased in NK cells in all tested cancer patients independently of tumor location, stage of disease or presence of metastases [7]. Our results suggested that altered signaling and expression of c-kit/SCF, c-myc and STAT3 in NK cells from cancer patients was responsible for the defect in NK cell cytolytic activity seen in many patients and that these abnormalities in the gene expression may be the cause rather than the result of tumor progression. 

NK cells play a key role in the induction and maintenance of anti*tumor* responses. *NK cells* directly kill *tumor cells* and release soluble factors that affect both innate and adaptive immunity. *NK cells* are also critically important for elimination of *tumor* metastases and probably dormant cancerous cells [8,9]. There is a clear correlation of the peripheral blood NK cell exhaustion state and the risk of cancer, although the exact mechanisms leading to NK cell exhaustion at the tumor milieu are poorly defined [10,11,12]. Considering significance of NK cells in antitumor immunity and their capability of killing malignant cells without prior sensitization, NK cells have been successfully tested for cell-based immunotherapy against cancers [13,14]. For instance NK cells can be genetically modified to express chimeric antigen receptors (CAR) in order to improve specific recognition of cancer surface markers [15]. Recent data confirming the importance of the inhibited NK cell functioning in vivo for cancer development and demonstrating that NK cells, in addition to T cells, mediate the effect of checkpoint blockade immunotherapy, reinforce our interests in NK cell-based cancer immunotherapy [16]. Although NK therapy is promising, many obstacles will need to be overcome, including understanding of actual mechanism of NK cell defects in tumor development and progression.

Here, we determined expression of both c-myc mRNA and protein expression in NK cells harvested from the peripheral blood of patients with lung and gastric cancer and correlated detected alterations with the defects in NK cell cycle and apoptosis development. 

Our data show that understanding the defects of oncogene functioning in immune cells in cancer should provide new markers for early cancer detection and accelerate the development of novel targeted therapies to destroy the stable and supportive cancer microenvironment. 

## 2. Results

### 2.1. Reduced c-myc mRNA Expression in NK Cells in Cancer Patients

**Figure 1:**
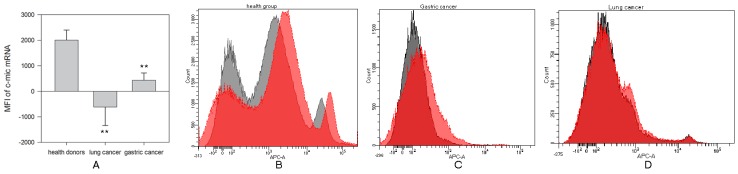
Differences in c-myc mRNA expression in NK cells harvested from healthy donors and cancer patients. NK cells were isolated from the peripheral blood samples by negative selection using Dynabeads, incubated in complete medium for 20 h and c-myc expression was determined by Smart Flare method as described in M&M. (**A**) Data of mean fluorescent intensity (MFI) are shown as the mean ± SEM (ANOVA). (**B**) C-myc-mRNA expression in peripheral NK cells from one of 10 representative healthy donors. (**C**) C-myc-mRNA expression in peripheral NK cells from one of 7 representative patients with lung cancer. (**D**) C-myc-mRNA expression in peripheral NK cells from one of 12 representative patients with gastric cancer. (**B**–**D**) The relative expression was determined by flow cytometry on stained NK cells.

Estimation of c-myc mRNA expression in the peripheral blood NK cells isolated from patients with lung cancer and gastric cancer was carried out by the Smart Flare method (Figure 1). No significant differences between patients with lung cancer or gastric cancer were identified. However, c-myc mRNA expression in NK cells from patients with lung cancer (−619 ± 724) and gastric cancer (430 ± 285) was significantly decreased compared with c-myc expression in NK cells from healthy donors (2004 ± 394) (** *p* < 0.002 and ** *p* < 0.004, respectively, Figure 1B–D). 

We noticed no highly significant association between c-myc mRNA expression and clinical stage of disease or the presence of metastases. However, expression of c-myc mRNA in NK cells from patients with well-differentiated (G1) and moderately differentiated (G2) types of carcinoma was commonly higher than one in NK cells from patients with poorly differentiated (G3) adenocarcinoma. The lowest values of the NK cell c-myc mRNA expression was determined, as a rule, in patients with poorly differentiated (G3) cancer.

Similar differences in c-myc expression were determined in NK cells isolated from patients after elective surgery. For instance, resection of tumor mass decreased the level of c-myc mRNA expression in NK cells in a patient with poorly differentiated (G3) carcinoma (MFI: 1020 before and 417 after operation). However, in a patient with well-differentiated (G1) type of tumor, one week after a surgical removal of the tumor mass, expression of c-myc mRNA in NK cells was doubled (MFI:1114 before and 3291 after operation). 

#### 2.1.1. Decrease in c-Myc Protein Production in NK Cells from Cancer Patients

**Figure 2:**
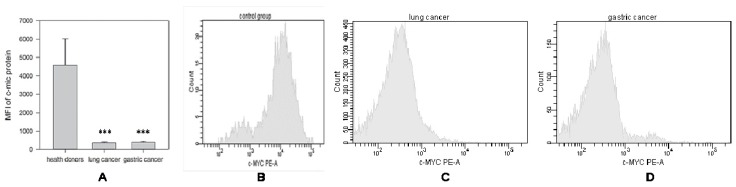
Differences in c-Myc protein expression in NK cells harvested from healthy donors and cancer patients. NK cells were isolated from the peripheral blood samples by negative selection using Dynabeads. C-myc-protein expression was determined as described in M&M. (**A**) Data of mean fluorescent intensity (MFI) are shown as the mean ± SEM (ANOVA). (**B**) C-myc-protein expression in peripheral NK cells from one of 10 representative healthy donors. (**C**) C-myc-protein expression in peripheral NK cells from one of 7 representative patients with lung cancer. (**D**) C-myc-protein expression in peripheral NK cells from one of 12 representative patients with gastric cancer.

Production of c-Myc protein was evaluated simultaneously with mRNA measurement in the same NK cells isolated from the same patients with lung cancer and gastric cancer (Figure 2). In contrast to c-myc mRNA data, the production of c-Myc protein was significantly reduced in all patients, regardless of the cancer cell differentiation stage, clinical stage of the disease or the presence of metastases (Figure 2A). For instance, c-Myc protein expression in the peripheral blood NK cells in patients with lung cancer (*n* = 7) (MFI: 375 ± 52) and gastric cancer (*n* = 12) (MFI: 406 ± 47) were similarly and significantly (*** *p* < 0.001 and *** *p* < 0.001, respectively) reduced when compared with expression of c-Myc protein in NK cells from healthy donors (*n* = 10) (MFI: 4574 ± 1446).

Interestingly, tumor resection did not recover c-Myc protein production by NK cells: c-Myc protein levels were quite low and stay low in all tested groups. Although mRNA expression of c-myc increased after surgery in patient with well-differentiated (G1) tumor stage, no significant changes in protein expression in NK cells were detected. For instance, MFI of c-myc protein expression before the surgery was 396 ± 45, while after surgery MFI increased up to 573 ± 78. The absence of a statistically significant correlation between the mRNA and protein data requires explanation, and additional mechanistical studies are in progress in our laboratories. 

#### 2.1.2. MAPK Activity in NK Cells from Cancer Patients

**Figure 3:**
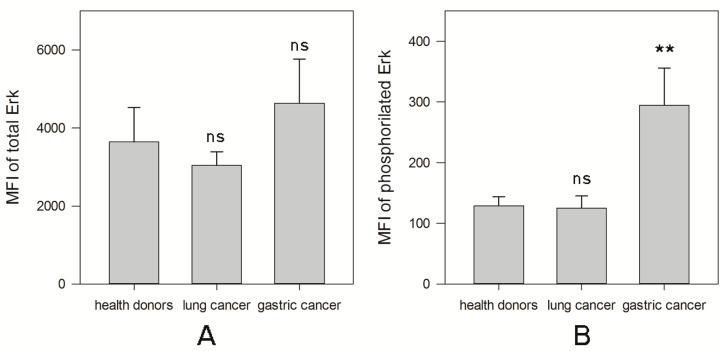
Differences in the total and phosphorylated level of ERK in NK cells harvested from healthy donors and cancer patients. NK cells were isolated from the peripheral blood samples by negative selection using Dynabeads. The total and phosphorylated levels of ERK were determined as described in M&M. (**A**) Data of MFI of total levels of ERK are shown as the mean ± SEM. (**B**) Data of MFI of phosphorylated levels of ERK are shown as the mean ± SEM. (** *p* < 0.01).

Many signaling pathways, including mitogen-activated protein kinase (MAPK), regulate *c-myc* mRNA expression and promote c-Myc protein stability in different cell types [17]. Reduction of c-Myc expression in NK cells may be associated with the deregulated ERK/MAPK (Extracellular signal-Related Kinase) activity as it was demonstrated that the inhibition of the MEK/ERK pathway dramatically decreased c-Myc expression in other cell types [18,19]. To test this, we have determined the ERK activation in different NK cell groups (Figure 3). No significant changes in the levels of total ERK protein in NK cells in any patient groups were detected (Figure 3A). However, there was a significant difference between two groups of patients in the levels of activated ERK. The level of phosphorylated protein in NK cells in patients with gastric cancer (294 ± 61) was higher than in NK cells isolated from healthy donors (129 ± 15) (** *p* < 0.01, Figure 3B). However, MAPK phosphorylation in NK cells in patients with lung cancer (125 ± 20) was similar to that one in NK cells harvested from healthy volunteers (“ns” *p* = 0.77). 

#### 2.1.3. Defect of Cell Cycle in NK Cells from Cancer Patients

**Figure 4:**
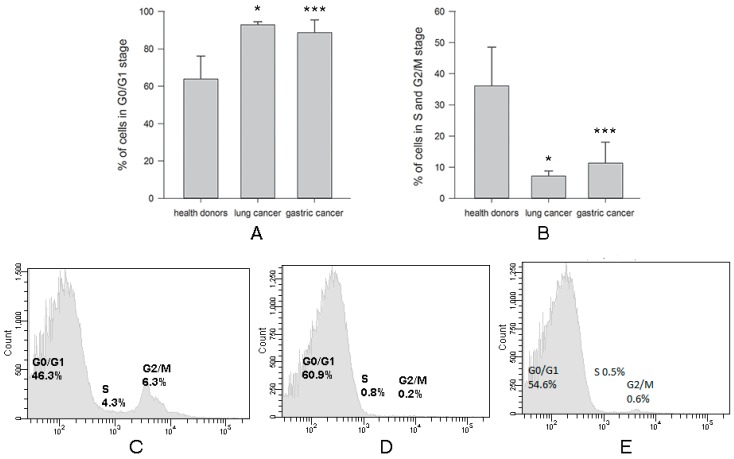
Differences in % NK cells in cell cycle phases harvested from healthy donors and cancer patients. NK cells were isolated from the peripheral blood samples by negative selection using Dynabeads. Cell cycle was determined as described in M&M. (**A**) Data of % NK cells in G0/G1 stage are shown as the mean ± SEM (ANOVA). (**B**) Data of % NK cells in S and G2/M stage are shown as the mean ± SEM (ANOVA). (**C**) Cell cycle of NK cells from one of 10 representative healthy donors. (**D**) Cell cycle of NK cells from one of 7 representative patients with lung cancer. (**E**) Cell cycle of NK cells from one of 12 representative patients with gastric cancer. (* *p* < 0.05 and *** *p* < 0.001).

The proto-oncogene *c-myc* encodes a transcription factor which plays a pivotal role in cell proliferation, metabolism, differentiation, and apoptosis by regulating expression of various downstream target genes. For example, cell cycle progression from the G0/G1 to the S phase is tightly controlled by c-Myc [20]. Therefore, we tested whether a decrease in expression of both c-myc mRNA and protein was associated with the cell cycle of NK cells. Our data demonstrated a highly visible mitotic arrest of NK cells from patients with both types of cancer in G0/G1 phase of the cell cycle. Numbers of NK cells from lung cancer (93 ± 2) (*n* = 7) and in gastric cancer (89 ± 7) (*n* = 12) in G0/G1 phase of the cell cycle were significantly increased (* *p* < 0.05 and *** *p* = 0.001, respectively) when compared with NK cells isolated from healthy volunteers, where the number of NK cells in this phase was 64 ± 12 (*n* = 10) (Figure 4A). Subsequently, the amount of NK cells isolated from all patients with lung and gastric cancer in phases G2/M and S decreased in comparison to controls: 7 ± 2 in lung cancer and 11 ± 7 in gastric cancer versus 36 ± 12 in healthy donors (* *p* < 0.05 and *** *p* = 0.001, respectively) (Figure 4B).

## 3. Discussion

Different means of surgical and non-surgical conventional treatment of cancer, including chemotherapy and radiation therapy, have not yielded completely satisfactory results to date. However, immunotherapy is in the center of attention of oncologists and clinicians as a treatment of choice for several types of cancer, although despite encouraging results of several clinical trials, clinical outcomes remain insufficient for most patients [21,22,23]. Despite of the successful therapeutic responses in patients with melanoma after checkpoint inhibitor treatment [24,25], the application of anti-PD-1 immunotherapy to several other cancers was less effective [18,19,26,27].

In this study, we not only confirm our early data related to an abnormal expression of the proto-oncogenes in cancer [7], but we also revealed changes in the expression of the c-myc proto-oncogene, both mRNA and protein, in NK cells in patients with lung and gastric cancer. A defect in the expression of both c-myc mRNA and protein should lead to metabolic cell cycle arrest. Indeed, our data showed that NK cells were in different stages of cell cycle, but they were not dead. It is unlikely that in such a sluggish state of cycling NK cells can display a feasibly activity against tumor cells. One can speculate this may serve as a mechanism of low cytotoxic activity of NK cells seen in cancer patients causing a low efficacy of antitumor protection [28,29,30,31]. Since the revealed defects of NK cells in cancer patients do not depend on stage of disease, localization, and metastatic spread, these defects rather precede cancer development than are the result of tumor growth. In line with this, recent “-omic” data (tumor RNA-seq (RNA sequencing) and whole-exome sequences of germline genomes) of The Cancer Genome Atlas of cancer patients (*n* = ~6,000) representing 13 common cancer types suggest that individuals who have inherited defects in NK cells experience a high risk of developing cancers [32]. Specifically, in all most common cancers, defects of the NK-specific-genes (i.e., NKD+ NK cell receptors) alone were sufficient to establish the critical correlations with patient survival and tumor infiltration by cytotoxic T cells. The authors speculate that inheritable defected of two key antitumor activities of NK cells—cytotoxicity and immune cell recruitment—results in low-efficient cancer immunosurveillance and high risk of tumor development and formation of metastases. The authors propose that “cancer is largely a disease of NK cell deficiencies” [32].

Our results demonstrating the independence of the revealed defects in NK cells from the localization of the tumor also suggest a common mechanism for the occurrence of immune defects during tumor development. Although this is supported by our and others’ data, further clinical and experimental confirmation is needed. Furthermore, more studies focusing on the oncogene expression and function in non-malignant immune cells are required to confirm their common or unique role in immunological defects in premalignant and malignant lesions. Although the defects of proto-oncogenes, in particular c-myc, are the attribute of many types of neoplasms with more than 50% of tumors overexpressing c-Myc protein [33], their role in controlling immune disfunctions in cancer is not well understood. We reported here that c-myc expression in NK cells, on the contrary to cancerous cells, decreased, while the low level of apoptotic death is a similar attribute in tested cells. Since, oncogenes such as Myc may up-regulate tumor development not only through their fundamental influence on cell proliferation but also via their control of immune checkpoints that enables evasion from immune surveillance [34], it will be interesting to validate whether Myc can regulate the expression of the immune checkpoint gene products in NK cells, as well as if other oncogenes, which are known to modulate Myc, can regulate immune checkpoints in NK cells. In line with this, targeting NK checkpoints to up-regulate their cytotoxic activity is now considered as a suitable approach for cancer treatment [35]. Modulation of NK numbers and/or function by a variety of agents such as cytokines (i.e., IL-15 and IL-2), monoclonal antibodies blocking inhibitory receptors (i.e., KIR, NKG2A and TIGIT (T cell immunoreceptor with Ig and ITIM domains) and agonists delivering signals via CD137, NKG2D and CD16 is widely discussed as the suitable therapeutic opportunities [35]. In addition to the approaches controlling inhibitory signals that limit NK cell function and redirecting NK cell activity against malignant cells, diverse approaches incorporating the development of large-scale NK cell-expansion protocols for adoptive transfer and the establishment of a microenvironment favorable to NK cell activity are now being undertaken to fully exploit NK cell antitumor properties in the clinic [36]. Finally, understanding the negative effect of the tumor microenvironment on NK cells in vivo, genetic modification of NK cells may deliver novel opportunities for developing successful cancer immunotherapies by improving NK cell responses and making them less vulnerable to the tumor microenvironment [37].

In conclusion, we revealed a significant reduction of both c-myc mRNA and protein in NK cells obtained from patients with lung and gastric cancer, which was independent from the stage of disease or the presence of metastases. Attenuated expression of c-myc was associated with lower number of dead NK cells and the mitotic arrest of NK cells in G0/G1 phase of the cell cycle. These data allow the suggestion of a new pathway of NK cell abnormality in cancer, which may provide new markers for early cancer detection or selecting patients with high risk of *carcinogenesis* or metastatic disease. 

The most important innovative potential of the manuscript is not only in confirmation and further expansion of unique recent findings of abnormal c-myc expression in NK cells isolated from patients with lung and stomach cancer, but in further development of clinically important data. This includes data validation on both the mRNA and protein levels, analysis of c-myc expression in cells on different stages of cell cycle and cell viability, and, most importantly, evaluation of c-myc expression in NK cells harvested before and after surgical tumor excision. Altogether, these new data offer a basis for a novel concept of tumor-associated immunosuppression and offer new targets in NK cells for development innovative and efficient cancer therapies.

## 4. Materials and Methods

### 4.1. Patients and Samples

Peripheral blood specimens were collected from 19 patients: 15 men and four women, median age 67 (39–84) with two types of cancer—lung cancer and gastric cancer. The studies were conducted in accordance with the Declaration of Helsinki, were approved by the Institutional Review Board (IRB) and patients provided written informed consent for sample acquisition for research purposes. Blood was collected prior to the surgical and chemotherapy procedures. Healthy controls (HC, *n* = 10, 5 men and 5 women, median age 54 (45–63)) were recruited from the personnel of the Laboratories within the Research Institute of Fundamental and Applied Medicine after signing of the informed consent. 

### 4.2. Purification of NK Cells

PBMC were isolated from the peripheral blood by Ficoll-Paque™ PLUS (Life Technologies, Waltham, MA, USA) density gradient centrifugation (Centrifuge 2-16k; Sigma, Darmstadt, Germany). NK cells were negatively selected using DynaMag™-5 Magnet with Dynabeads^®^ Untouched™ Human NK Cells isolation kit (Life Technologies). The purity of the cell subsets was confirmed by flow cytometry (FACSCalibur, BD Biosciences, San Jose, CA, USA) using CD56 and CD16 monoclonal antibodies labeled with FITC (Fluorescein isothiocyanate) and PerCP (BD Biosciences) as described earlier [7]. The purity of isolated NK cells was typically between 90–95% and potential contamination with NKT (Natural Killer T cells) cells was less than 1–2% according to the manufacturer procedure.

### 4.3. SmartFlare™ RNA Detection Assay

Isolated NK cells were incubated in RPMI-1640 medium supplemented with 10% FCS in 96-well flat-bottom plates at 37 °C, 5% CO_2_ for 20 h in the presence of Smart Flare Myc, Human, Cyanine 5 according to the manufacturer’s instructions (Merck Millipore, Burlington, MA, USA). Control probes were incubated with: (i) Cellular uptake control; (ii) Scramble control for specificity and (iii) Housekeeping control the 18s gene. Fluorescence was detected by flow cytometry on BD FACSCantoTM II.

### 4.4. Detection of c-Myc Protein Production

Isolated NK cells were fixated, permeabilizated, washed, and then mixed with fluorochrome-conjugated antibodies (Human c-Myc PE-conjugated Antibody, R&D Systems, Minneapolis, MN, USA). Fluorescence was detected by flow cytometry on BD FACSCantoTM II. 

### 4.5. MAPK Activation Dual Detection Assay

The levels of both the total and phosphorylated protein were measured simultaneously in the same cells using Millipore’s FlowCellect™ MAPK Activation Dual Detection kit. This Kit includes two directly conjugated antibodies: the phospho-specific anti-phospho-ERK1/2 (Thr202/Tyr204, Thr185/Tyr187)-PE and anti-ERK1/2-Alexa Fluor^®^ 647-conjugated antibody to measure the total levels of ERK. This two-color flow cytometry kit allows measuring the extent of MAPK phosphorylation relative to the total MAPK expression in cells. Cells were fixated, permeabilizated, washed, and then mixed with fluorochrome-conjugated antibodies. Fluorescence was detected by flow cytometry on BD FACSCantoTM II.

### 4.6. Cell Cycle Assay

Tali^®^ Apoptosis Kit containing Annexin V Alexa Fluor^®^ 488 and 7 amino actinomycin-D was used for detection of early and late stages of apoptosis in tested cells. 7-AAD (7 aminoactinomycin D) is a membrane impermeant dye that is excluded from viable cells and so shows the dead cell count. The number of dead cells did not exceed 1%. Fluorescence was detected by flow cytometry on BD FACS CantoTM II. 

### 4.7. Statistical Analysis

For a single comparison of two groups, the Student’s *t*-test was used after the evaluation of normality. If data distribution was not normal, a Mann–Whitney rank sum test was performed. For the comparison of multiple groups, analysis of variance was applied. SigmaStat Software was used for data analysis (SyStat Software, Inc.). For all statistical analyses, *p* < 0.05 was considered significant. All experiments were repeated at least two times. Data are presented as the mean ± SEM.
